# Policy analysis of nutrition stewardship for prevention and control of Non-communicable diseases in Iran

**DOI:** 10.1186/s12913-023-09087-2

**Published:** 2023-01-25

**Authors:** Mohammad Amerzadeh, Amirhossein Takian, Hamed Pouraram, Ali Akbari Sari, Afshin Ostovar

**Affiliations:** 1 Social Determinants of Health Research Center, Research Institute for Prevention of Non-Communicable Diseases, Qazvin university of Medical Sciences, Qazvin, Iran; 2 Department of Health Management, Policy & Economics, School of Public Health, Tehran University of Medical Sciences (TUMS), Tehran, Iran; 3 Department of Global Health and Public Policy, School of Public Health, Tehran University of Medical Sciences (TUMS), Tehran, Iran; 4 Heath Equity Research Center (HERC) – TUMS, Tehran, Iran; 5 National Center for Health Insurance Research, The Iranian Health Insurance Organization, Tehran, Iran; 6 Community Nutrition Department, School of Nutritional Sciences and Dietetics, Tehran University of Medical Sciences (TUMS), Tehran, Iran; 7 Osteoporosis Research Center, Endocrinology and Metabolism Clinical Sciences Institute, Endocrinology and Metabolism Research Institute, Tehran University of Medical Sciences (TUMS), Tehran, Iran

**Keywords:** NCDs, Nutrition, Government, Stewardship, Iran

## Abstract

**Background:**

Non- communicable diseases (NCDs) are the main cause of death, which lead to over 73% of death and 62% of DALYs globally. As an unhealthy diet is the leading behavioral risk factor of NCDs, in line with the national action plan for the prevention and control of NCD, this study explored the nutrition-related stewardship problems to reduce the burden of NCDs in Iran.

**Methods:**

This is a qualitative study. We interviewed 30 purposefully identified key informants, i.e., stakeholders, policymakers, and academics, from December 2018 to August 2019. All interviews were recorded and transcribed verbatim. We analyzed data using qualitative content analysis facilitated by MAXQDA 11 software.

**Results:**

Ample policies and laws were identified, most of which were not or partially implemented. Despite some plausible efforts, NCDs do not seem to be a top priority for high-level managers and decision-makers. Besides, the role of non-state actors, i.e., the private sector, is marginal in NCD’s planning and management. Whereas the government, e.g., the Food and Drug Organization (FDO), is the biggest player. Worse still, many harmful products are advertised and easily distributed across the country.

**Conclusion:**

Iran’s government has created a noticeable roadmap to battle NCDs despite imposing many sanctions and related socioeconomic problems. Nevertheless, more interventions are needed to strengthen the stewardship of NCDs by various stakeholders. We recommend the government to monitor the implementation of policies and advertisement of harmful products to prioritize prevention and control of NCDs. In addition, we advocate employing the capacity of non-state actors to reduce the consumption of unhealthy food and the burden of NCDs across the country, ultimately.

## Background

Non- communicable diseases (NCDs) are the main cause of death, leading to over 73% of death and 62% of disability-adjusted life years (DALYs) globally [1]. 78% of whole NCD deaths and 85% of premature global death due to NCDs occur in low- and middle-income countries (LMICs). An adult in the LMIC has a doubled risk of death from NCDs compared to his counterpart from a high-income country (HIC) [2]. With over 82% of deaths attributed to NCDs, Iran has a high burden of NCDs [1, 3].

Dietary risks factors, i.e., increasing consumption of processed food containing high amounts of sugar, salt, and trans fats, with the low consumption of healthy foods, e.g., fruits and vegetables, nuts and pulses, are the leading behavioral risk factor for NCDs and related death worldwide [4]. Improvement in diet might prevent 20% of deaths globally [5, 6]. Annually, billions of US dollars are allocated to advertise foods containing high fat, sugar, and salt, and their intake has risen worldwide [5]. In Iran, one study found that a significant percentage of the current food commercials advertise unhealthy diets or nutritionally questionable diet products [7].

In response to the emerging burden of NCDs, the World Health Organization (WHO) provided the global action plan for the prevention and control of NCDs (2013–2020), which comprises nine voluntary targets in different aspects, including lowering the consumption of unhealthy diet [8]. In line with this and the WHO best buys, in 2015, Iran approved its national action plan for the prevention and control of NCDs, including various interventions for diverse levels of governance at national, subnational, and municipal councils to reduce the burden of NCDs. It included a 30% reduction in salt intake until 2030 and zero trans-fatty acids by 2020 in food & oily products [3, 9, 10].

Stewardship is “the administrator’s willingness and ability to earn public trust by being an effective and ethical agent in carrying out the republic’s business.” Stewardship is a governance model infusing policy-making and regulatory functions via an explicitly normative dimension [11]. Stewardship is a central building block of any health system. It might affect policy-making at different levels, especially at higher levels, influencing the quality of healthcare services [12]. Dietary risk factors are among the primary cause of NCDs in Iran. Therefore, Iran’s government has implemented diverse interventions to improve unhealthy diets and decrease salt, sugar, and fat consumption, and consequent mortality related to NCDs [13]. Our previous study shows that Iran’s status of NCDs ‘policies based on best buys recommendations is generally advance. We developed a repository of Iran’s policy documents about salt, sugar, and fat and conducted content analysis and interviews with pertinent stakeholders. We categorized policies into three parts: red color (lack of policy documents), amber (inspirational policy without action), and green (policy in operation). Our analysis revealed that 20 policies were green, one amber, and no policy was red [13]. Aiming to improve policy-making for better management of NCDs, we report the stewardship of nutrition in Iran as the fundamental pillar to success. Studies on the nutrition-related stewardship problems in the LMICs are scarce. To the best of our knowledge, this is the first study in Iran to identify nutrition-related stewardship problems in terms of policies and laws. Our findings can help policymakers achieve the targets in the national action plan for prevention and control of NCDs, specifically SDG 3.4, to reduce 30% of premature death due to NCDs by 2030 in Iran and probably similar settings.

## Methods

### Study design and data collection

This is a qualitative study. We collected data via 30 semi-structured, face-to-face interviews with purposefully identified experts, including policymakers, top-level managers, academics, and other stakeholders, from December 2018 until August 2019 in Tehran. We developed and applied a generic interview guide, provided participants with an information sheet, obtained informed consent, and reassured them about data confidentiality and anonymity. The interviews were digitally recorded, transcribed verbatim, and thematically analyzed.

## Sampling

Purposeful and snowball sampling methods were used to choose the participants. We continued the interviews until we reached saturation. Initially, we reviewed documents and reports to select key informants and organizations. Subsequently, we identified and added other stakeholders introduced by the key informants of the previous step. We tried to select the participants from diverse, relevant disciplines and related to diet and NCDs from four groups: policymakers, healthcare services providers, and regulatory organizations, academics. They include various departments affiliated with the Ministry of Health and Medical Education (MoHME), Ministry of Agriculture, Ministry of Industry, Mine and Trade, Ministry of Education, Municipalities, National Standard Organization, Ministry of Economic Affairs and Finance, Islamic Republic of Iran Broadcasting (IRIB), and Planning and Budget Organization. Table 1 presents the characteristics of the study participants. First, we had a personal contact or called the participants’ office and then set a time to conduct the interviews.

**Table 1:** The characteristics of interviewees

Sector	Number of participants
Different Departments at the MOHME	9
Schools of Nutrition in Universities of Medical Sciences	2
Iranian Academy of Medical Sciences (AMS)	1
Food and Drug Organization (FDO)	3
Supreme Council for Health and Food Security (SCHFS)	2
Ministry of Agriculture	1
Ministry of Industry, Mine and Trade	1
Ministry of Education	1
Deputy of Public Health at the Universities of Medical Sciences	2
Municipalities	2
National Standard Organization	1
Ministry of Economic Affairs and Finance	1
Islamic Republic of Iran Broadcasting (IRIB)	1
WHO Office in Iran	1
Planning and Budget Organization	1
Non-Communicable Diseases Research Center affiliated with Tehran University of Medical Sciences (TUMS)	1

### Data analysis

We carried out the qualitative content analysis using deductive and inductive approaches in data analysis, facilitated by MAXQDA 11 software^1^ [14, 15]. We analyzed the data in five phases, i.e., familiarization, identification of the thematic framework, indexing, mapping, and interpretation. The first and corresponding author carried out the categorization process, and other authors revised and approved the entire steps. Two researchers also checked the coding system to enhance the validity. Finally, we sent some transcripts, categories, subcategories, and codes to selected participants to obtain their approval, which helped us increase the interpretations’ accuracy and credibility [16, 17].

### Ethical considerations

The Ethics Committee of Tehran University of Medical Sciences (TUMS) approved this study (the ethical code: IR.TUMS.REC.1397.193). All methods were carried out in accordance with relevant guidelines and regulations.

## Results

We conducted 30 interviews, including five policymakers, 13 top-level managers, eight participants from regulatory organizations, and four academics. Table 2 summarizes the identified themes and sub-themes (one theme and five sub-themes) related to the stewardship of prevention and control of NCDs in Iran. The categories include incomplete and inconsistent implementation of laws, plans, and policies; lack of insight about prevention and control of NCDs in other sectors; structural weakness of non-state actors; insufficient actions in population-based interventions; and advertising harmful products.

**Table 2:** Themes and sub-themes related to the stewardship of prevention and control of NCDs in Iran

Theme	Category	Examples of the barriers
Stewardship factors	Incomplete and inconsistent implementation of laws, plans, and policies	Selling unhealthy sea salt, false claims about its benefits, and overlooking its likely side effects in advertisements. The subsidy system deviated from its initial purposes; the lack of a system to impose a tax on unhealthy products.
	Lack of insight into the prevention and control of NCDs in other sectors	Actors outside the MoHME generally have inadequate insight into the prevention and control of NCDs
	The structural weakness of non-state actors	NGOs do not have a powerful position in the prevention and control of NCDs; Inadequate utilization of the private sector
	Insufficient actions in population-based interventions	Industry-based interventions are appropriate, while population-based interventions are not efficient enough
	Advertising harmful products	Allocating advertisement codes to the harmful products
		.

Table 2**: Themes and sub-themes related to the stewardship of prevention and control of NCDs in Iran.**

- **Incomplete and inconsistent implementation of laws, plans, and policies**

Despite ample policies and laws, the participants identified none or incomplete implementation of such plans related to the consumption of an unhealthy diet as a significant concern. For example, some experts promote using sea salt on TV and in national media, misunderstanding that it is healthier and has no side effects. There are some false claims about its benefits, and its likely side effects are overlooked in advertisements:

*“The problem is not the existence of policy or law; it is the non-implementation of laws, such as selling unhealthy and dirty salt of the sea, claimed to have side effects. When an expert proposes a law in other countries, orders are given to the food industry, and they must implement them.” (PMN12).*

In addition, when laws and policies are approved, they need an executive guarantee, action plans, and some monitoring to be followed up, which is a gap in some sectors:

“*For example, laws have been passed to reduce traffic accidents in Iran. These laws are sent to us in the MoHME to be followed up. But most do not have an executive guarantee, and the law is not implemented.” (HEN18).*

Some interviewees complained about the inefficient implementation of the Targeted Subsidies Law, which aimed to create more jobs and assist industries in promoting public health:

*“In the Targeted Subsidies Law, our goal was to improve the nutritional pattern and access to a healthy diet. But it was deflected, and the diet pattern did not change as expected.” (HEN13).*

Worse still, the inappropriate tax system did not lead to the increasing price of unhealthy products and hindered the promotion of healthy foods:

*“We were not successful in implementing tax policies because it needs the law and the cooperation of other sectors. Unfortunately, we did nothing positive in this regard, even taxing cigarettes.” (Policy maker 26).*

- **Lack of insight into NCDs prevention and control in other sectors**

Despite some plausible efforts, NCDs do not seem to be a top priority for high-level managers, decision-makers, and actors outside the MoHME. At the same time, NCDs are a severe issue in the country and lead to many deaths:

“*When we need other sectors’ cooperation like the Ministry of Youth Affairs and Sports, they express that their priority is championship sports, not public sports and control of NCDs*.” (HEN18).

In general, the priority of the organizations at different levels, such as the consumer and the producer, should be NCDs, not just the MoHME’s priority:

*“In other sectors, there is no insight into NCDs prevention. I talk to the agriculture sector, and I cannot have their cooperation because they have a production-based insight, not the health and NCDs.” (PMN1).*

- **The structural weakness of non-state actors**

One strategy to implement policies and interventions, especially in issues related to health and promoting a healthy diet, is NGOs and delegating part of the government’s power to them. However, in the context of Iran, many NGOs are governmental organizations, which are sponsored by the government, and their existence depend on governmental support. It decreases their powerful position in the prevention and control of NCDs:

*“This politician has nothing to do with the people. The government does not share its power with people and NGOs. As a result, the NGOs have no power in the country’s governance structure, and they are under government control.” (HEN24).*

Another strategy to implement policies and interventions is to pay special attention to the private sector as a critical stakeholder. Nonetheless, the private sector’s potential, like the food industries and voluntary interventions, has not been utilized enough in NCD’s planning and management in Iran. It looks like that the government does not take the private sector as a critical player in policymaking:

*“The government has all the tools, but there is a problem in practice in which the country is under heavy sanctions and factories have to deal with many pressures, and it ends up to the government’s more active role.” (HEN11).*

- **Insufficient actions in population-based interventions**

Many experts believed that the FDO’s measures taken to reduce the consumption of unhealthy food are good enough, and industry-based interventions are appropriate, e.g., food labeling interventions, while population-based interventions were branded inefficient:

*“One side of the issue is that the FDO itself has implemented legal tools for food and pharmaceutical industries and companies. But there is not enough tool for society and the people in practice. “(PMN10).*

One interviewee stated:

*“We need to talk to people about reducing consumption of unhealthy diet convincingly and patiently and highlight the role of it in the health of families and society. We should do the same with organizations related to governance, including the agriculture, industry sector, and other stakeholders.” (HEN6).*

- **Advertising harmful products**

One major problem with harmful products is the Ministry of Culture and Islamic Guidance’s advertising centers, which supervise all the advertisements in the country. The advertising center is not allowed to advertise until it receives an advertising code from the Ministry of Culture and Islamic Guidance for each product. According to the approvals of the SCHFS, the task force comprising representatives of the MoHME must approve the advertising content of food products before broadcasting in the media. However, the practice is different, and many harmful products are advertised and easily distributed across the country:

*“Each advertisement must have the approval of the Ministry of Culture and Islamic Guidance. If this Ministry does not permit it, they will not advertise it, like advertisements against cultural issues. It is impossible to see any ad in the city contrary to our cultural, customary, and religious issues.”(PMN18).*

Besides, there is a parliamentary act in which the FDO can prevent the advertisement of harmful products, while their promotion is also prohibited at the discretion of the FDO. Nevertheless, the act is not fully implemented:

*“We have passed a single article in the parliament that the FDO can address the promotion of harmful products. It has enough power to control misleading advertising and food that should not be advertised.” (PMN26).*

One interviewee revealed the reason for not implementing this law in some cases:

*“The industries sometimes control media. The media has power and money, and because the MoHME does not have much money, they could not involve in media much.” (PMN10).*

Further, public health literacy is generally low regarding the need to consume less harmful products, hence the need to enhance public awareness through appropriate training:

*“Some people express that if a product is harmful, why is it licensed? Consumption of harmful products should not become a habit. People should have the right to choose because many of these goods are nutritionally harmful and not toxic. If we consume less, there is no problem.” (PMN26).*

## Discussion

This article studied the stewardship-related factors affecting healthy nutrition in Iran. Our studies revealed that there are ample policies and laws, most of which are not or partially implemented, and the stakeholders do not cooperate reasonably. Another study in Iran reviewed the rules and regulations and concluded that there are enough legislation and programs to improve food security schemes in Iran [18]. To bridge the implementation gap, the study called for further intersectoral cooperation to program and monitor plans [18]. A study in Denmark also found that coordinated actions by the government and other stakeholders to ban the use of trans fatty acids in food between 1976 and 2005 caused the most significant decline in trans fatty acid consumption per capita (5.4 g per day) [19]. Hence, the need to meaningfully engage with various stakeholders to properly implement the rules.

Despite some plausible efforts, according to our participants, NCDs do not seem to be a top priority for high-level managers, decision-makers, and actors outside the MoHME. A similar study revealed that in continuation of the current pattern in NCDs, Iran would not be able to reach SDG 3.4. It shows that Iran has a downward trend in NCDs mortality, which will continue by 2030; Nevertheless, this trend is not enough to achieve SDG 3.4 [20]. Paying more attention to intersectoral cooperation, particularly the SCHFS structure, and appropriate governmental support are key to involving other sectors. Another study found that implementing population-based policies, i.e., NCDs prevention and control, needs strong national high-level leadership, even at the head of State (in Iran, president), to foster multi-sectoral collaboration by involving diverse non-health sectors [21, 22]. Another study emphasized that Iran, as a nation with a rising rate of NCDs and limited resources, needs to consider and prioritize the best policies to control the possible devastating outcomes [23].

In addition, non-state actors, i.e., NGOs and the private sector, have been conventionally marginalized in Iran. Evidence shows the need to involve the private sector in producing and promoting healthy food consumption, which is among the key measures for preventing and controlling NCDs [24]. Another study highlights the importance of the private sector in the LMICs in helping governments towards the prevention and control of NCDs [25]. Recognition and enthusiasm for involving the private sector is the initial move to work with corporations. The private sector has the capability to attract its business and scientific skills and focuses on robust results-based performance. Governments are also expected to facilitate the private sector’s participation in prevention and control of NCDs, through adpting appropriate strategies at the national level, i.e., adjusting taxes and subsidies [26]. Besides, NGOs are influential media to address global problems such as the environment, peace, and poverty [27]. More public-private partnerships with NGOs can utilize resources more effectively to address NCDs [28]. Due to the weak intersectoral capacities to initiate and strengthen meaningful actions, extra support from the public sector, civil society, media, and NGOs are essential to succeed in the whole population interventions for prevention and control of NCDs [29]. Therefore, we advocate NGOs’ meaningful engagement to increase the effectiveness of activities for NCDs program.

Further, despite the FDO’s good initiatives to reduce the consumption of unhealthy food, more population-based interventions are required to foster strong, quick, equitable, and cost-saving measures for effective prevention and control of NCDs in Iran [30], e.g., enhancing health literacy through social media and campaigns [31] to reduce unhealthy diet and harmful products’ advertisement. One study in Australia revealed that watching more advertisements heightens interest and the tendency to consume harmful products like sweets, fast food, and soft drinks [32, 33]. Another study found that the unhealthy products industries are the key drivers of global NCD epidemics. Therefore, evidence-based measures such as legislation, regulations, tax policies, pricing, and limitation of advertising are helpful in decreasing the death and disability related to NCDs [34]. One Iranian study identified the gap in implementing a parliamentary law that bans advertisements of harmful products across all media and called upon executive organizations to enforce and control the law [35].

The Targeted Subsidies Law was launched in 2010 to improve equity in Iranian society. It was expected to allocate the targeted subsidies to create more jobs and promote public health and healthy products. In practice, it faced many obstacles because of flawed formulation, inappropriate implementation, and unregulated evaluation [36]. Worse still, the weak multi-sectoral collaboration prevented the meaningful execution of imposing a sin tax on harmful products while subsidizing healthy goods, which aims to encourage people in the lower socioeconomic groups to take more healthy products [36].

### Rigor of study

To our knowledge, this is the first study in Iran to identify problems related to nutrition stewardship in Iran. We were not able to convince some interviewees to take part in our research, in spite of our utmost efforts. Nevertheless, the in-depth interviews with diverse stakeholders let us collect a rich data source. Further, we carried out this study before the COVID-19 pandemic. Meanwhile, people’s diets might have changed for several reasons, e.g., economic conditions and food accessibility, particularly among vulnerable citizens. Our analysis is contextual-based and applicable to the Iranian community’s characteristics. Caution is necessary for generalizing far-reaching conclusions from our study.

## Conclusion

Despite all odds, i.e., unilateral sanctions and economic difficulties, the government of Iran has created a noticeable roadmap to battle NCDs. Our study reindorses the need for the government to monitor the implementation of policies and advertisement of harmful products to prioritize prevention and control of NCDs. In addition, we advocate employing the capacity of non-state actors to reduce the consumption of unhealthy food and the burden of NCDs across the country. In line with the SDG 3.4 to reduce 30% of premature death due to NCDs and related risk factors by 2030 in Iran, strong and effective stewardship is the key to fuel all building blocks of the health system and equip it with enough resources, policies, expertise, and leverage to reduce the consumption of unhealthy food and ultimately the burden of NCDs across the country.

## Data Availability

The datasets used and/or analyzed during the current study are available from the corresponding author on reasonable request. The entire dataset is in the Farsi language. The data can be available in the English language for the readers and make available from the corresponding author on reasonable request.

## Abbreviations

NCDs: Non- communicable diseases
DALYs: disability-adjusted life years
FDO: Food and Drug Organization
WHO: World Health Organization
MoHME: Ministry of Health and Medical Education
IRIB: Islamic Republic of Iran Broadcasting
TUMS: Tehran University of Medical Sciences

## Footnotes

1 https://www.maxqda.com
